# Electrochemical and X-ray Photoelectron Spectroscopy Surface Characterization of Interchain-Driven Self-Assembled Monolayer (SAM) Reorganization

**DOI:** 10.3390/nano12050867

**Published:** 2022-03-04

**Authors:** Angelo Tricase, Anna Imbriano, Nicoletta Ditaranto, Eleonora Macchia, Rosaria Anna Picca, Davide Blasi, Luisa Torsi, Paolo Bollella

**Affiliations:** 1 Dipartimento di Chimica, Università degli Studi di Bari Aldo Moro, 70125 Bari, Italy; angelo.tricase@uniba.it (A.T.); anna.imbriano@uniba.it (A.I.); nicoletta.ditaranto@uniba.it (N.D.); rosaria.picca@uniba.it (R.A.P.); davide.blasi@uniba.it (D.B.); paolo.bollella@uniba.it (P.B.); 2 Centre for Colloid and Surface Science, Università degli Studi di Bari Aldo Moro, 70125 Bari, Italy; 3 Dipartimento di Farmacia-Scienze del Farmaco, Università degli Studi di Bari Aldo Moro, 70125 Bari, Italy; eleonora.macchia@uniba.it; 4 Faculty of Science and Engineering, Åbo Akademi University, 20500 Turku, Finland

**Keywords:** self-assembled monolayers, cyclic voltammetry, X-ray photoelectron spectroscopy, conformational rearrangements, single-molecule detection

## Abstract

Herein, we report a combined strategy encompassing electrochemical and X-ray photoelectron spectroscopy (XPS) experiments to investigate self-assembled monolayer (SAM) conformational reorganization onto an electrode surface due to the application of an electrical field. In particular, 3-mercaptopriopionic acid SAM (3MPA SAM) modified gold electrodes are activated with a 1-ethyl-3-(3-dimethylaminopropyl)carbodiimide (EDC) and *N*-hydroxysulfosuccinimide (NHSS) (EDC-NHSS) mixture by shortening the activation time, from 2 h to 15/20 min, labelled as Protocol-A, -B and -C, respectively. This step, later followed by a deactivation process with ethanolamine (EA), plays a key role in the reaction yields (formation of *N*-(2-hydroxyethyl)-3-mercaptopropanamide, NMPA) but also in the conformational rearrangement observed during the application of the electrical field. This study aims at explaining the high performance (i.e., single-molecule detection at a large electrode interface) of bioelectronic devices, where the 3MPA-based SAM structure is pivotal in achieving extremely high sensing performance levels due to its interchain interaction. Cyclic voltammetry (CV) experiments performed in K_4_Fe(CN)_6_:K_3_Fe(CN)_6_ for 3MPA SAMs that are activated/deactivated show similar trends of anodic peak current (I_A_) over time, mainly related to the presence of interchain hydrogen bonds, driving the conformational rearrangements (tightening of SAMs structure) while applying an electrical field. In addition, XPS analysis allows correlation of the deactivation yield with electrochemical data (conformational rearrangements), identifying the best protocol in terms of high reaction yield, mainly related to the shorter reaction time, and not triggering any side reactions. Finally, Protocol-C’s SAM surface coverage, determined by CV in H_2_SO_4_ and differential pulse voltammetry (DPV) in NaOH, was 1.29 * 10^13^ molecules cm^−2^, being similar to the bioreceptor surface coverage in single-molecule detection at a large electrode interface.

## 1. Introduction

The chemical modification of surfaces represents a key step in the development of (bio)sensors [1,2,3,4,5,6,7,8]. In this regard, interchain interactions (e.g., Van der Waals interactions, hydrogen bonds, etc.) play a key role in the control of growth kinetics and SAM conformational rearrangements [9,10,11,12]. For thiol-based SAMs, the formation of the typical comb-like structure proceeds via a two-step process, where high-energy interchain interactions like hydrophobic forces [13,14,15,16,17], dipole-dipole interactions [18], and hydrogen bonds [10,11,19], can lead to complex SAM reorganisation within the densely packed monolayer [9,11,20,21,22,23]. Hence, the optimization of SAM layers growth protocols is crucial for device development [24,25,26], with interchain interactions largely affecting their performance [13,27,28]. In particular, SAMs bearing carboxylic groups undergo a two-step reaction encompassing the activation performed in 1-ethyl-3-(3-dimethylaminopropyl)carbodiimide (EDC) and *N*-hydroxysulfosuccinimide (NHSS) (EDC/NHSS) mixture at slightly acidic pH (4.8–5.6), resulting in the formation of a fairly stable sulfo-NHS ester. Afterwards, the blocking/deactivation was performed by reacting the latter with ethanolamine (EA), generating *N*-(2-hydroxyethyl)-3-mercaptopropanamide (NMPA) onto the sensing surface. It should be highlighted that the EDC/NHSS mixture remains active only for 15 min, while sulfo-NHS ester has a half-life of 4–5 h at pH 7, 1 h at pH 8, and only 10 min at pH 8.6 [29,30,31]. Hence, long activation time might result in a plethora of side reactions, generating several by-products (e.g., anhydride formation, acidic hydrolysis, intermolecular reactions, etc.) [32,33,34], affecting the analytical figures of merit of a sensing device [35,36,37,38,39,40,41,42,43,44,45,46,47].

Indeed, a more relevant role has been hypothesised when implemented in electrolyte gated organic field effect transistors (EGOFETs); biosensors, described by Macchia et al., exhibited an LOD at the single-molecule level, with 60 zM in 100 µL of sample volume [48,49,50], considering a millimetre-sized sensing interface modified with 10^11^–10^12^ bioreceptor cm^−2^ (*wide-field* approach) [48,49]. Herein, diffuse interchain hydrogen bondings between amides and carboxylic groups within the SAM monolayer play a key role in the sensing mechanism, hypothesising a signal amplification, which is fundamental for single-binding event detection, which is still under investigation.

In this regard, electrochemical, morphological, and spectroscopic methods can provide several insights on supramolecular/conformational rearrangements. Electrochemical techniques, such as cyclic voltammetry (CV) and electrochemical impedance spectroscopy (EIS), are typically used for evaluating SAM conformational rearrangements, their resistance to the charge transfer under different conditions, and electron transfer kinetics on redox-active groups embedded in the chain structure [35,51,52,53,54]. Morphological techniques such as atomic force microscopy (AFM) and scanning-tunnelling microscopy (STM) are widely used to estimate the surface homogeneity and morphology [55,56,57,58], whereas grazing-angle attenuated total reflectance (GA-ATR) infrared spectroscopy and X-ray photoelectron spectroscopy (XPS) are used for molecular and elemental speciation, providing chemical information about functional groups and elements enclosed in the SAM structure [59,60,61].

In previous reports [32], the ratio between amides and carboxylic acids was evaluated, considering both activation and deactivation steps (later defined as the deactivation protocol). The final NH/COOH ratio resulted in approximately 60%, with a slightly higher value for Protocol-B (55 ± 8% for Protocol-A, 63 ± 7% for Protocol-B). Later, the same authors elucidated the SAM supramolecular structure using IR spectroscopy and investigated its conformational rearrangement through electrochemical measurements [62]. NMPA SAMs showed a peculiar rearrangement, forming a tight structure when exposed to the electric field. This behaviour is probably related to high-energetic chain interaction characteristics for similar SAMs, leading us to further explore amide SAMs in a more complex configuration.

In this paper, we report the electrochemical characterization of 3MPA SAMs, shortening the activation time (reaction of -COOH groups with EDC/NHSS mixture at slightly acidic pH, as previously defined) from two hours to 15/20 min, namely protocol-A, -B, and -C, to elucidate the effect on SAM conformational rearrangements while applying an electrical field. X-ray photoelectron spectroscopy (XPS) was used to compare the aforementioned activation protocols. Differential pulse voltammetry (DPV) in NaOH 0.5 M was used to evaluate the surface coverage of the most effective protocol, which was protocol-C [63]. In addition, a theoretical analysis of the data was also carried out. To this end, the SAM-modified electrodes exposed to a negatively charged redox probe solution were described using the interpenetration/diffusion model, reported in reference [62]. These results allow us to deepen the knowledge of SAM supramolecular/conformational rearrangements in order to design and optimize the performance of bioelectronic devices embedding the SAM structure, particularly contributing to the unravelling of the sensing mechanism behind such high performance for EGOFET-based biosensors.

## 2. Materials and Methods

### 2.1. Chemicals

3-Mercaptopropionic acid (3MPA), N-hydroxysulphosuccinimide (NHSS), 1-ethyl-3-(3-dimethylaminopropyl)carbodiimide (EDC), sodium hydroxide (NaOH), and ethanolamine chloride (EA) were purchased from Merck and used without further purification. A 0.1 M 2-(N-morpholino)ethane-sulfonic acid (MES) buffer (Sigma–Aldrich) solution was adjusted with NaOH 1 M at pH 4.8–4.9. Phosphate-buffered saline (PBS) solution (phosphate buffer of 10 mM, KCl 2.7 mM, NaCl 137 mM, pH 7.4) was prepared by dissolving a PBS tablet (Sigma–Aldrich) in 200 mL of HPLC water. The solution was filtered on a Corning 0.22 μm polyethersulfone membrane before use. Potassium Ferrocyanide (K_4_[Fe(CN)_6_]), Potassium Ferricyanide (K_3_[Fe(CN)_6_]), and sulfuric acid (H_2_SO_4_, 95–98%) were purchased from Sigma–Aldrich (now Merck). Solutions used for sample functionalization were prepared using HPLC water (Fluka/C. Erba), while solutions for electrochemical measurement (H_2_SO_4_, NaOH) were prepared in Milli-Q water (18.2 MΩ cm^−1^, Millipore, Bedford, MA, USA).

### 2.2. Electrode Preparation

Gold samples were prepared starting from an Si wafer covered by a thermally grown 0.3 μm thick SiO_2_. Firstly, Si:SiO_2_ substrates (Si-Mat, Germany) were cleaned by subsequent sonication in HPLC water, acetone, and isopropanol (10 min for each step) and then dried under N_2_ flux. Finally, a 5 nm thick Ti adhesion layer and a 50 nm thick gold film were e-beam evaporated. Both steps were performed through a shadow mask. The active layer consisted of a 20 mm^2^ circular area; for the substrate configuration at end of the process, please refer to [62,63]. After the e-beam evaporation samples were sonicated in an apolar solvent (n-heptane) for 10 min, they were immersed in a Piranha Solution (H_2_O_2_:H_2_SO_4_ 3:7 *v*/*v*) for 5 min, then in boiling water for 10 min, and finally exposed to an Ozone Plasma for 10 min. Immediately afterward, the ozone-cleaning substrates were immersed in a 10 mM 3MPA solution for 20 h. To prevent thiol group oxidation, ethanol was degassed under N_2_ for 10 min before thiol solution preparation. Moreover, SAM growth was performed under N_2_ atmosphere and in amber vials to limit oxygen diffusion and light exposition.

**Figure 1:**
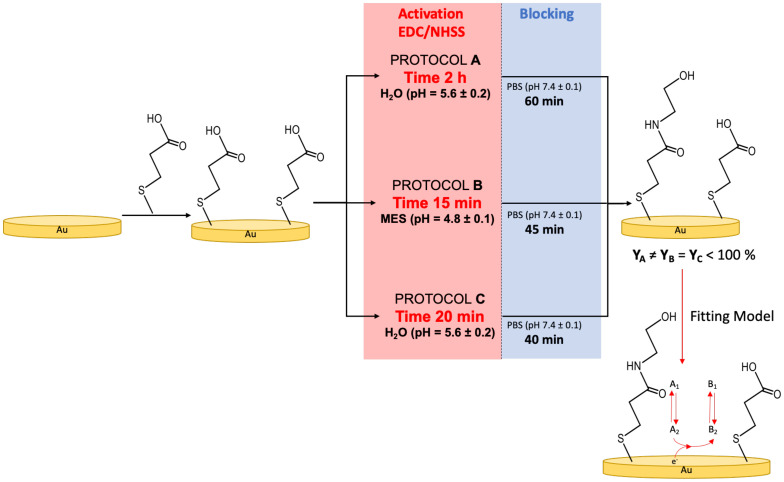
Scheme of SAM growth and deactivation protocols. Data were fitted using the interpenetration/diffusion model reported in reference [62] and summed up on the last panel (Fitting model).

3MPA SAMs were prepared following three different protocols, addressed as protocol-A, protocol-B, and protocol-C. Protocol-A consisted of an exposition to an EDC/NHSS solution (0.2 M/0.05 M) in HPLC water for 2 h (activation step), followed by an immersion in a 1 M EA solution in PBS for 60 min (blocking step). Protocol-B involved the same steps, but the activation was carried out for 15 min in MES buffer (pH 4.8 ± 0.1), and the blocking time was equal to 45 min. Protocol-C was a combination of Protocol-A and -B, using the same solvent as Protocol-A (HPLC water for activation and PBS for blocking), but with a step- time length similar to Protocol-B (20 min for activation, 40 min for blocking). Protocol-A, -B, and -C are described in Figure 1.

Hereafter, samples functionalized using these protocols are indicated as follows:

1. Deactivated SAMs, without specifying the protocols used.
2. Protocol-A SAMs, Protocol-B SAMs, and Protocol-C SAMs, for describing an SAM functionalised using a specific protocol.

### 2.3. Electrochemical Measurements

Electrochemical measurements were carried out using a CH1230b potentiostat-galvanostat. A conventional three-electrode electrochemical cell was used for the electrochemical experiments, consisting of an Ag/AgCl (KCl 3 M) as the reference electrode (RE) (all the potential values in this work are reported vs. this reference), a platinum wire as the counter electrode (CE), and a gold electrode, eventually modified with SAMs, as the working electrode (WE). Cyclic voltammetry (CV) measurements were performed in two different solutions: (a) in a 1:1 solution of K_4_[Fe(CN)_6_] and K_3_[Fe(CN)_6_] in PBS (1 mM total), and (b) in H_2_SO_4_ 0.5 M. The measurements were performed using a potential window ranging from −0.3 to 0.6 V for CVs in K_4_[Fe(CN)_6_]:K_3_[Fe(CN)_6_], and from 0.5 to 1.7 V for CVs in H_2_SO_4_. All the measurements were carried out at a 100 mV s^−1^ scan rate. Starting potential was set at the lower potential value. CVs in K_4_[Fe(CN)_6_]:K_3_[Fe(CN)_6_] were performed leaving samples in the cell for a maximum of 210 min, periodically acquiring the voltammograms (after 0, 20, 30, 60, 90, 120, and 210 min from the beginning of the experiment). SAM reductive desorption was performed using a Differential Pulse Voltammetry (DPV) in NaOH 0.5 M, as previously reported in the literature [64]. The conditions included an initial potential of −0.6 V, a final potential of −1.3 V, an increment potential of 8 mV, a pulse amplitude of 25 mV, and a pulse width of 0.1 V. All data were analysed using OriginPro 2021.

### 2.4. Electrochemical Model

Ion permeation/diffusion in an SAM-modified electrode was described using a model reported in a previous paper [51]. The process is described as a three-step reaction, where the redox molecule was modelized as a generic molecule A (oxidized form) or B (reduced form). The molecule A diffuses from the solution bulk (A_1_) to the electrode surface (A_2_) and then reacts, forming the reduced form (B_2_), which can diffuse from the electrode surface to the solution bulk (B_1_). This chemical-electrochemical-chemical (CEC) process is schematically reported in Figure 1.

### 2.5. X-ray Photoelectron Spectroscopy (XPS) Analyses

X-ray photoelectron spectroscopy analyses were performed with a Versa Probe II Scanning XPS Microprobe spectrometer (Physical Electronics GmbH, Feldkirchen, Germany). Samples were prepared using protocols reported in the sample preparation section. The measurements were carried out with a monochromatized AlKα source (X-ray spot 200 µm) at a power of 49.2 W. Wide scans and detailed spectra were acquired in fixed analyser transmission (FAT) mode, with a pass energy of 117.40 eV and 46.95 eV. An electron gun was used for charge compensation (1.0 V 20.0 μA). All binding energies were referenced to the C-C component of C1s at 284.8 ± 0.1 eV for adventitious carbon and cross-checked with the Au4f_7/2_ component at 84.0 ± 0.1eV. Data processing was performed using MultiPak software v. 9.9.0.8, 2018 (ULVAC-PHI, Inc., Hagisono, Chigasaki, Kanagawa, Japan) [65].

## 3. Results and Discussion

### 3.1. Cyclic Voltammetry Analyses on Au-SAMs

**Figure 2:**
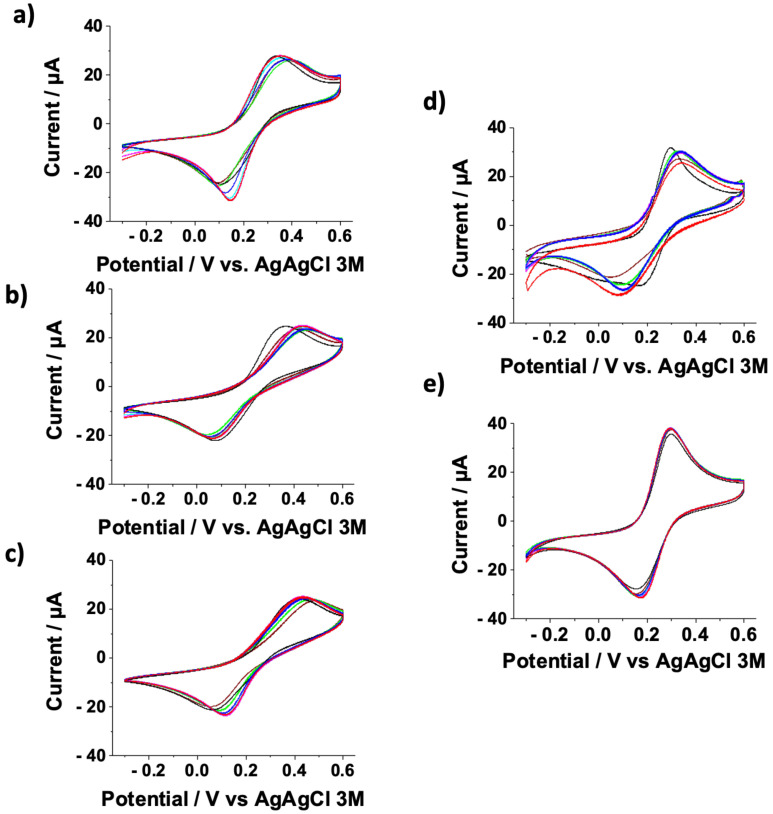
Overlapped reiterated cyclic voltammetry for (**a**) Protocol-A SAM, (**b**) Protocol-B SAM, (**c**) Protocol-C SAM, (**d**) 3MPA SAM, and (**e**) bare Au in K_4_Fe(CN)_6_:K_3_Fe(CN)_6_ (1 mM total). Experimental conditions: 10 mM PBS buffer pH 7; scan rate 100 mV s^−1^ and T = 25 °C. Each panel reports curves acquired after 0, 20, 30, 60, 90, 120, and 210 minutes from the beginning of the experiment. Samples were left in the electrochemical cell for the whole measurement time.

To evaluate SAM conformational rearrangement, several CVs were carried out using bare and 3MPA SAM-modified gold electrodes (as negative controls) and gold electrodes modified with 3MPA SAMs undergoing the following activation/deactivation protocols: Protocol-A, Protocol-B, and Protocol-C (as displayed in Figure 1). Notably, 3MPA SAMs bear -COOH functional groups, while deactivated SAMs have both -NH_2_ and -COOH moieties, probably because of incomplete surface activation [32,33,34]. Both SAM systems exhibited a diffuse hydrogen bonding. However, deactivated SAMs showed a stronger interchain hydrogen bonding interaction, involving both -NH_2_ and -COOH groups [10,19]. The -NH_2_/-COOH ratio in Protocol-A and Protocol-B was around 3:2 [32]: 54 ± 7% and 64 ± 8%, respectively. Moreover, Protocol-C was similar to Protocol-B. SAM conformational rearrangements occur under the effect of an electrical field, mostly depending on the polarity of functional groups [62,66]. In this regard, to evaluate the rearrangement kinetics, samples were left in a 1:1 solution of K_4_Fe(CN)_6_:K_3_Fe(CN)_6_ (1 mM total) in PBS for 210 min and characterized by performing a CV scan after 0, 20, 30, 60, 90, 120, and 210 min from the beginning of the experiment. In Figure 2, CVs for (a) Protocol-A SAM, (b) Protocol-B SAM, (c) Protocol-C SAM, (d) 3MPA SAM, and (e) bare gold are reported. In the three deactivation protocols (Figure 2a–c), the anodic peak current (I_A_) in the range 25–30 μA remains unchanged, probably because of the hydrogen bonding interchain interactions driving the SAM conformational rearrangements. The latter elicits the interpenetration/diffusion and electron transfer through pinholes of negatively charged redox probe at the electrode surface. In the negative controls, it was possible to observe the decreasing of I_A_ over time for 3MPA SAM-modified electrodes (Figure 2d), where the reorganisation of short negatively charged -COO^–^ ended chains hindered the interpenetration/diffusion and electron transfer phenomena. Moreover, the bare gold electrode (Figure 2e) did not show any I_A_ variation because of a permanent pinhole-like scenario (I_A_ values in the range 35–38 μA). In fact, the elicited interpenetration/diffusion model describes the electrochemical measurement as a three-step reaction, where the first and the last reaction account for the interpenetration/diffusion of the redox probe between the solution bulk and electrode surface [51]. In a similar scenario, the SAM layer acts as hindering barrier, limiting the redox probe diffusion onto the electrode surface.

**Figure 3:**
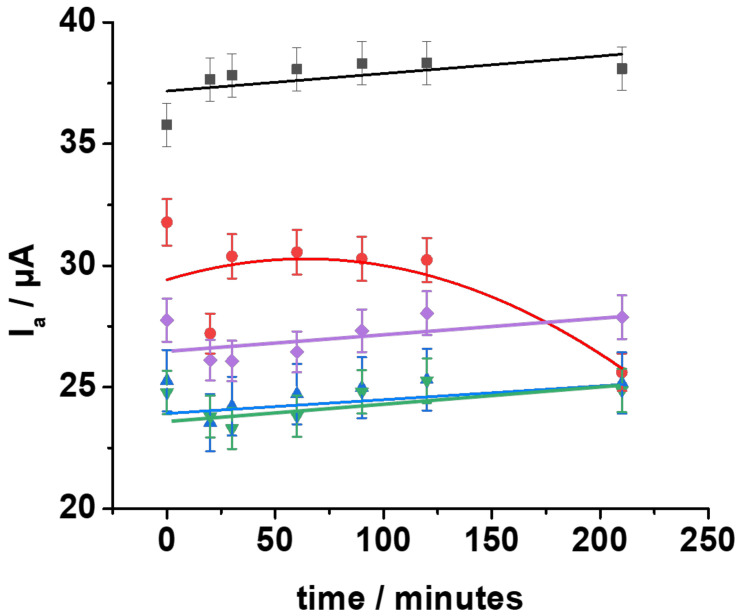
Anodic peak currents over time measured for gold (black line), 3MPA SAM (red line), Protocol-A SAM (purple line), Protocol-B SAM (green line), and Protocol-C SAM (blue line). Experimental conditions are the same as reported for Figure 2. The fitting was performed using the model reported in [62].

Furthermore, I_A_ values vs. time for all electrode configurations are reported in Figure 3. All data were fitted using a combination of Butler–Volmer and Nicholson–Shain equations, as previously reported [62]. Deactivated SAMs (Figure 3, Protocol-A (purple curve), Protocol-B (green curve), and Protocol-C (blue curve)) showed a decreasing current after 30 min (5–6%), followed by a slower increase between 30–120 min and a steady state between 120–210 min. The I_A_ trend over time can be ascribed to the presence of amides (~60% of chains), increasing the interchain interaction energy [10,19,67] (hydrogen bonding), which hinders the formation of pinholes within the SAM structure, where ion penetration/diffusion and electron transfer usually occur [68]. Conversely, 3MPA SAMs (Figure 3, red curve) showed a slight decrease from 32 to around 30–31 μA in the first 120 min, followed by a remarkable decrease between 120 and 210 min (26 μA), which is similar to trends reported for aliphatic SAMs, where a high current decrease was reported for a long relaxion time [62]. Indeed, 3MPA SAMs are characterized by a diffuse hydrogen bonding but a relative low chain length. The latter limits hydrophobic interaction between chains, decreasing the stability of compact structures and promoting the formation pinholes and defects, which results in preferential pathways for the redox probe diffusion. Bare gold electrodes exhibited a steady-state trend because of a permanent pinhole-like scenario. The electrochemical results herein unequivocally prove that the conformational rearrangements of deactivated SAMs driven by strong interchain interactions (notably hydrogen bonding -NH/-COOH) play a key role in the single-molecule sensing performed with EGOFET-based biosensors [48]. Despite deactivated SAMs exhibiting similar trends, Protocol-A’s SAM showed a slightly higher I_A_ compared to Protocol-B‘s SAM and Protocol-C’s SAM, which should have had a similar structure according to our hypothesis. To confirm these differences, X-ray photoelectron spectroscopy was used to compare the deactivated SAMs.

### 3.2. X-ray Photoelectron Spectroscopy (XPS) Characterization of Conformational Rearrangements of SAM Supramolecular Structure

X-ray photoelectron spectroscopy (XPS) measurements were carried out on all the samples previously described to support the electrochemical results. The high surface sensitivity of XPS makes it an ideal tool to study the chemical composition of the very short 3MPA SAMs investigated herein, along with that of their ending moieties. XPS elemental atomic percentages for all the samples are reported in Table 1.

**Figure 4:**
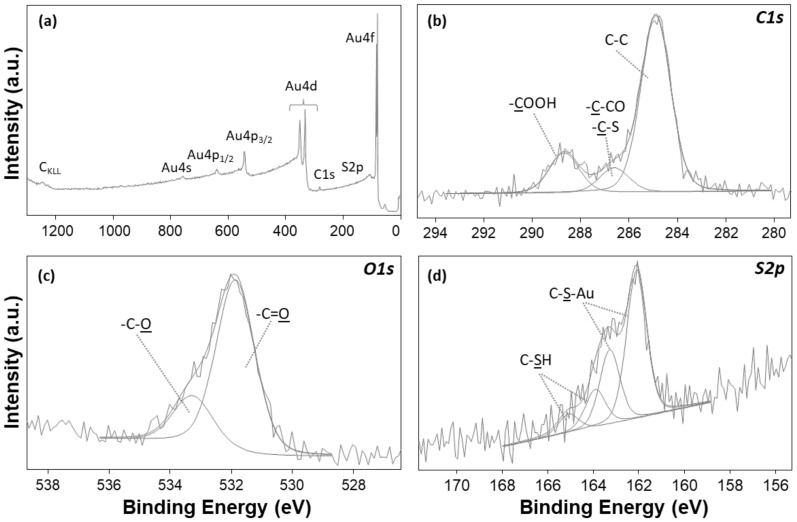
(**a**) XP survey spectrum of bare gold, with elemental signal assignments; (**b**–**d**) 3MPA SAM high-resolution spectral regions. High-resolution spectra are reported with the curve-fitting results and peak component attributions.

Bare gold was analysed to confirm the homogeneity of the Au layer. Au was the most abundant element (73.8 ± 1.1%). C1s and O1s signals were attributed to the natural surface hydrocarbon contamination of all air-exposed samples. The absence of other species, such as Si or SiO_2_, ascribable to the gold underneath the substrate, confirmed the uniformity of the Au layer thickness (Figure 4a). Afterwards, XPS analysis of 3MPA SAM-modified gold electrodes displayed the presence of C, O, and S. Notably, C1s and O1s curve-fitting results showed COOH and C-S peak components (Figure 4b–c). The S2p XP spectrum (Figure 4d) presented two doublets: the more abundant one, ascribable to covalently bound thiolate-Au (BE_S2p3/2_ = 162.0 ± 0.2 eV), and a second one, attributed to physiosorbed thiolate (BE_S2p3/2_ = 163.7 ± 0.2 eV) [69,70]. This evidence confirms the successful growth of carboxylic ending SAMs.

**Figure 5:**
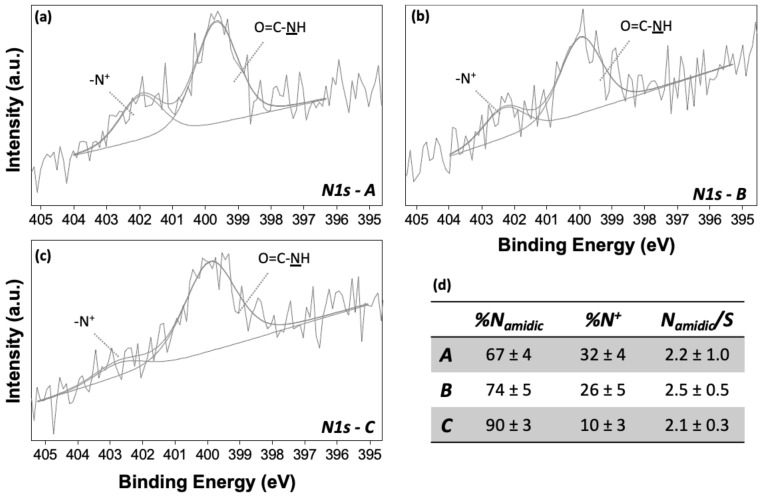
(**a**–**c**) N1s XP spectra of deactivated SAMs (Protocol-A, -B, and -C), reported with the curve-fitting results and peak component attributions. (**d**) Nitrogen component relative abundance and derived N_amidic_/S atomic ratio for Protocol-A, -B, and -C.

Deactivated SAMs presented a significant percentage of nitrogen (~3%). The curve fitting procedure applied to N1s signals resulted in two different components at 399.8 ± 0.2 and 402.5 ± 0.3 eV, compatible with amidic nitrogen and N^+^, respectively (Figure 5a–c). The former was attributed to the reaction of ethanolamine with activated carboxylic moieties, as elucidated in the scheme of Figure 1, while N^+^ was probably related to either the adsorption of residual ethanolamine salt formation or the degradation of unreactive species during the activation step [33]. Indeed, its percentage is higher for Protocol-A (more than 30% of the total nitrogen), demonstrating that longer activation time (2 h) can promote side reactions (Figure 5a) due to intra-chain rearrangement of EDC-terminating SAMs.

N_amidic_/S atomic ratio (Figure 5d), derived from nitrogen component relative abundance, was used for evaluating the deactivation protocol yield: in fact, each reacted chain presents one amide group for each sulphur atom, whereas unreacted chains have one sulphur atom and no amidic groups [33,34]. For Protocol-A, the yield was equal to 0.5 ± 0.2, and for -B and -C, to 0.7 ± 0.2. Protocol-A and -B yields confirmed the data gathered with the infrared study reported in reference [32], where a yield close to 60% was found, with a lower value for Protocol-A (56 ± 7%) than Protocol-B (65 ± 8%). Moreover, the similar yield found for Protocol-B and -C, characterized by similar time-length steps but different buffers for the activation step, confirmed the crucial role of the length of the step in carboxylic acid activation, as hypothesized in [32]. For this reason, Protocol-C resulted the best trade-off between reaction yield and convenient procedure, as water was used in place of the MES for the activation step. Therefore, Protocol-C was selected for further experimental investigation, as described in the next section. Cascade overlapped C1s, O1s, N1s, and S2p high-resolution spectral regions for all samples are reported in Figure S1.

### 3.3. Surface Coverage Evaluation

To evaluate the surface coverage, reductive desorption of Protocol-C SAMs was performed. To this aim, four different measurements were carried out: (a) CV in H_2_SO_4_ on bare gold to calculate the electroactive area of the electrode (Figure S2); (b) differential pulse voltammetry (DPV) in NaOH 0.5 M on bare gold, as the standard (Figure 5, red curve), (c) DPV under the same conditions of Protocol-C’s SAM (Figure 5, black curve), and (d) XPS on Protocol-C’s SAM after the reductive desorption (Figure S3 and Table 2).

**Figure 6:**
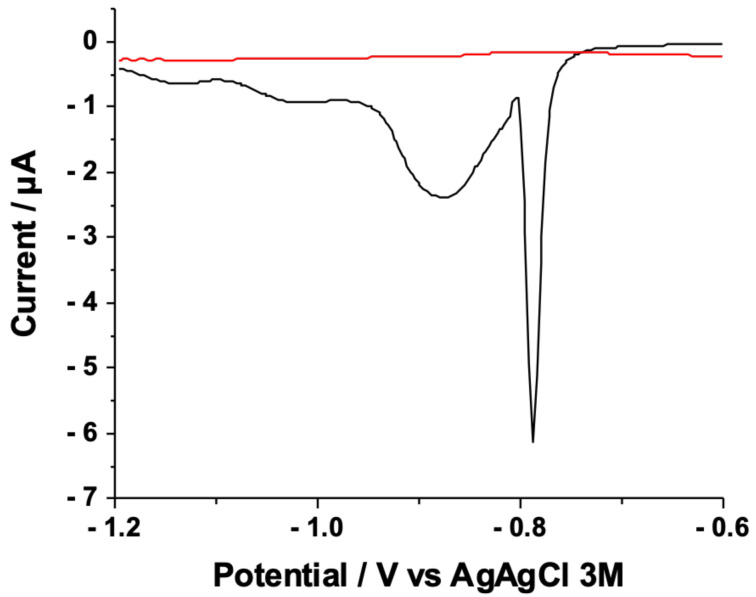
Differential pulse voltammetries (DPVs) on gold (red curve) and Protocol-C SAM (black curve) in 0.5 M NaOH. Experimental conditions: scan range −0.6 V to −1.2 V in reverse scan mode, scan rate 100 mV s^−1^.

In Figure S2, the gold oxide reduction peak (measured in H_2_SO_4_, vide infra) was used to measure the real electroactive area of gold, comparing the charge involved in this process (51 μC) with the theoretical charge density reported by Trasatti et al. (390 μC cm^−2^) [71]. The resulting area was 13 mm^2^, 65% of the geometrical area, which is comparable to the electroactive area previously reported [62]. In Figure 6, DPVs carried out from −0.6 V and −1.2 V for bare gold (red curve) and Protocol-C’s SAM (black curve) were reported. Protocol-C’s SAM voltammogram (black curve) showed a peak at −0.9 V, compatible with the SAM desorption process [64]. The sharp signal at −0.8 V can be ascribed to contaminants in the thiol solution. No signals were recorded on the voltammogram for the gold electrode (black curve). The reaction occurring during the DPV for Protocol C’s SAM was [72]

(1) $$RS-Au+e^{-}\to RS+Au$$

To confirm the complete desorption of the SAMs, XPS measurements were carried out after DPVs and compared to the data reported in the previous section. XPS elemental atomic percentages after SAM desorption are reported in Table 2.

The S2p region showed no signals after the desorption (sulphur amount below the limit of detection), while a very low intensity signal was present in the N1s region (%N was around 1.6 ± 1.4% compared to 2.8 ± 0.7% before the voltammetry (Table 1)). This evidence, along with the absence of -COOH components in C1s spectrum (data not shown), confirmed the effective desorption of SAMs. At this stage, surface coverage Γ can be measured by the following equation [73]:

(2) $$\Gamma =\frac{Q}{nFA}$$

where *Q* is the charge transferred during the reduction reaction, equal to the area of peak at −0.9 in the DPV in Protocol-C’s SAM, *n* is the number of exchanged electrons (notably one), *F* is the Faraday Constant, and *A* is the electroactive of the electrode calculated before (13 mm^2^). Surface coverage was 2.15 × 10^−11^ mol cm^−2^, equal to 1.29 × 10^13^ molecules cm^−2^, compatible with the results obtained by the Molecule Dynamic Simulation performed in previous papers, where surface coverage was in the order of 10^14^ molecules cm^−2^ for a similar system [48].

## 4. Conclusions

In this work, three different deactivated SAMs were compared using cyclic voltammetry and X-ray photoelectron spectroscopy, unequivocally proving that the application of electrical field (sensing measurements with EGOFETs) elicits SAM conformational rearrangements due to strong hydrogen bonding interchain interactions -NH/-COOH. Among the deactivated SAMs, based on the reaction yields (previously determined), we were able to observe different electrochemical behaviours towards interpenetration/diffusion and electron transfer processes occurring at the electrode. Protocol-A, -B, and -C showed a 5–6% decrease in the first 30 min, followed by an increase between 30–120 min and a steady state from 120 to 210 min. However, Protocol-A’s SAM, activated for two hours, showed higher I_A_ values, probably due to the presence of activation by-products (e.g., anhydrides, intramolecular rearrangements, etc.). These can limit the strength of interchain interactions matching with the limited amount of -NH groups on the electrode surface. Protocol-B’s SAM, activated for 15 min, exhibited slightly lower I_A_ values than Protocol-A’s SAM, matching the conformational rearrangements driven by hydrogen bonding interchain interactions -NH/-COOH, typical for higher reaction yields. Protocol-C’s SAM displayed similar electrochemical behaviour compared to Protocol-B’s SAM, probably due to comparable activation times (15 and 20 min for Protocol-B and -C, respectively), but slightly different pH, which is not effective for preventing side reactions. X-ray photoelectron spectroscopy confirmed our hypothesis, showing yields comparable with the ones previously reported for Protocol-A, -B, and -C [32]. Hence, Protocol-C resulted in the most efficient modification strategy, reporting a surface coverage of 1.29 × 10^13^ molecules cm^−2^. This value is comparable with results reported for similar systems embedded in EGOFET biosensors, enhancing the knowledge of SAM electrochemical behaviour as exploited for sensing purposes.

## Figures and Tables

**Table 1:** XPS relative atomic percentages for bare Au, 3MPA SAMs, Protocol-A SAM, Protocol-B SAM, and Protocol-C SAM. The % are reported as mean values ± 1S (*n* = 3).

	%C	%N	%O	%S	%Au
Bare Au	18 ± 2	n.d.	8.4 ± 1.9	n.d.	73.8 ± 1.1
3MPA SAM	36 ± 3	n.d.	12.7 ± 1.1	3.1 ± 0.5	48 ± 4
Protocol A SAM	30 ± 1	3.3 ± 1.7	17 ± 4	4.6 ± 0.5	45 ± 4
Protocol B SAM	32.1 ± 0.5	3.1 ± 0.5	18 ± 3	3.7 ± 0.5	43 ± 3
Protocol C SAM	29 ± 5	2.8 ± 0.7	14.8 ± 1.0	3.1 ± 0.5	50 ± 6

**Table 2:** XPS relative atomic percentages for Protocol-C SAM after reductive desorption. The % are reported as mean values ± 1S (*n* = 3).

	%C	%N	%O	%S	%Au
Protocol C SAM after reductive desorption	23.3 ± 1.4	1.6 ± 1.4	3.9 ± 1.7	n.d.	71 ± 2

## Data Availability

Publicly available datasets were analyzed in this study. This data can be found here: https://zenodo.org.

## Supplementary Materials

The following supporting information can be downloaded at: https://www.mdpi.com/article/10.3390/nano12050867/s1, Figure S1: Cascade overlapped C1s, O1s, N1s and S2p high resolution spectral regions for the samples reported in colour coded legend; Figure S2: Bare Gold cyclic voltammetry in H_2_SO_4_; Figure S3: S2pXP spectra Protocol-C SAM.
