# Supplementary material for: Electrochemical and X-ray Photoelectron Spectroscopy Surface Characterization of Interchain-Driven Self-Assembled Monolayer (SAM) Reorganization
Source: Nanomaterials (Basel). 2022 Mar 4;12(5):867. doi: 10.3390/nano12050867 (PMC8912756; doi:10.3390/nano12050867)
Supplement: Supplementary file 1 [file nanomaterials-12-00867-s001.zip › nanomaterials-1599958-supplementary.pdf]

Supplementary Materials

Electrochemical and X-ray Photoelectron Spectroscopy Surface Characterization of Interchain-Driven Self-Assembled Monolayer (SAM) Reorganization

Angelo Tricase [1,†], Anna Imbriano [1,2†], Nicoletta Ditaranto [1,2], Eleonora Macchia [3,4], Rosaria Anna Picca [1,2], Davide Blasi [1], Luisa Torsi [1,2,4,*] and Paolo Bollella [1,2]

1 Dipartimento di Chimica, Università degli Studi di Bari Aldo Moro, 70125 Bari, Italy; angelo.tricase@uniba.it (A.T.); anna.imbriano@uniba.it (A.I.); nicoletta.ditaranto@uniba.it (N.D.); rosaria.picca@uniba.it (R.A.P.); davide.blasi@uniba.it (D.B.); paolo.bollella@uniba.it (P.B.)
2 Centre for Colloid and Surface Science, Università degli Studi di Bari Aldo Moro, 70125 Bari, Italy
3 Dipartimento di Farmacia-Scienze del Farmaco, Università degli Studi di Bari Aldo Moro, 70125 Bari, Italy; eleonora.macchia@uniba.it
4 Faculty of Science and Engineering, Åbo Akademi University, 20500 Turku, Finland
* Correspondence: luisa.torsi@uniba.it
† These authors contributed equally to this work.

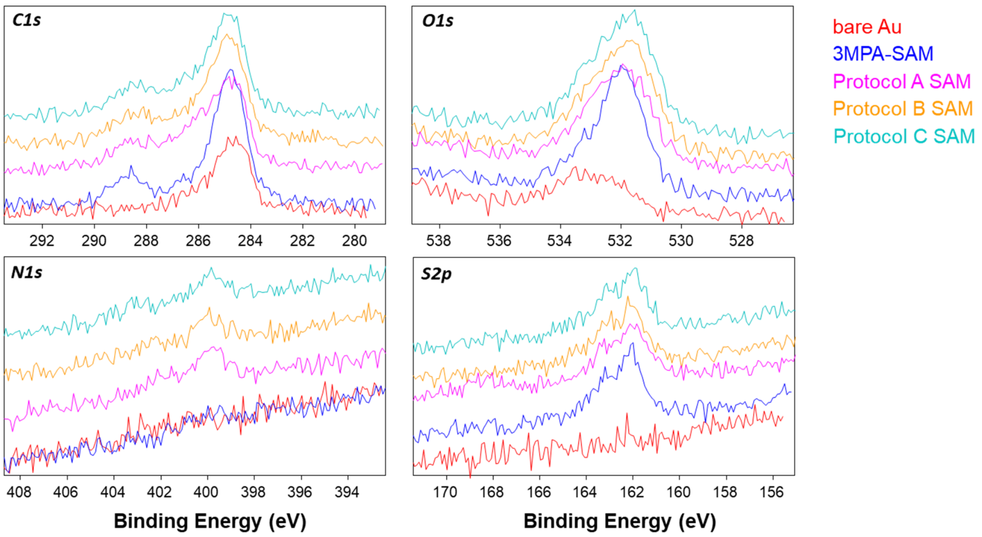

Figure S1. Cascade overlapped C1s, O1s, N1s and S2p high resolution spectral regions for the samples reported in colour coded legend.

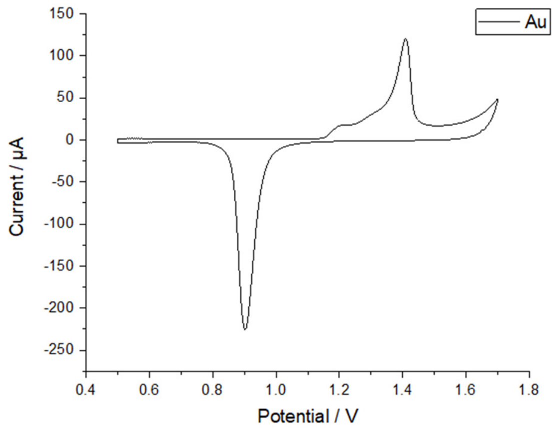

Figure S2. Cyclic voltammetry of bare Au in H_2SO_4 0.5 M. Experimental conditions: scan range 0.5-1.8 V in positive direction, scan rate 100 mV s^{-1}, T=25°C. Peak in cathodic scan between 0.9 and 1.1 V is attributed to the Au reductive peak and proportional to the Au electroactive area [1].

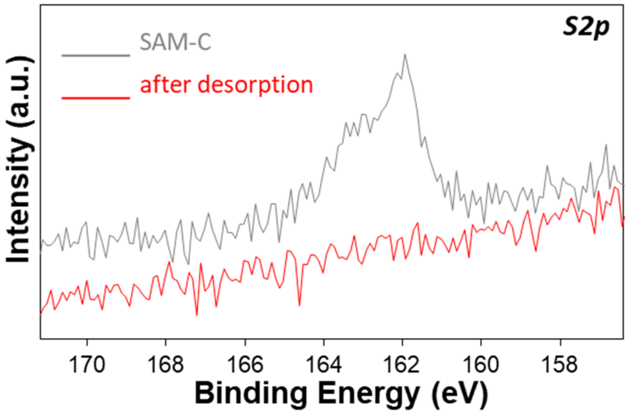

Figure S3. S2p XP spectra Protocol-C SAM before (grey) and after (red) the reductive desorption.
